# Assessing risk of fibrosis progression and liver-related clinical outcomes among patients with both early stage and advanced chronic hepatitis C

**DOI:** 10.1371/journal.pone.0187344

**Published:** 2017-11-06

**Authors:** Monica A. Konerman, Dongxia Lu, Yiwei Zhang, Mary Thomson, Ji Zhu, Aashesh Verma, Boang Liu, Nizar Talaat, Ulysses Balis, Peter D. R. Higgins, Anna S. F. Lok, Akbar K. Waljee

**Affiliations:** 1 University of Michigan Health System, Division of Gastroenterology and Hepatology, Ann Arbor, Michigan, United States of America; 2 University of Michigan Health System, Department of Internal Medicine, Ann Arbor, Michigan, United States of America; 3 University of Michigan Health System, Division of Pathology, Ann Arbor, Michigan, United States of America; 4 VA Ann Arbor Health Services Research and Development Center of Clinical Management Research, Ann Arbor, Michigan, United States of America; University of North Carolina at Chapel Hill School of Medicine, UNITED STATES

## Abstract

**Objective:**

Assessing risk of adverse outcomes among patients with chronic liver disease has been challenging due to non-linear disease progression. We previously developed accurate prediction models for fibrosis progression and clinical outcomes among patients with advanced chronic hepatitis C (CHC). The primary aim of this study was to validate fibrosis progression and clinical outcomes models among a heterogeneous patient cohort.

**Design:**

Adults with CHC with ≥3 years follow-up and without hepatic decompensation, hepatocellular carcinoma (HCC), liver transplant (LT), HBV or HIV co-infection at presentation were analyzed (N = 1007). Outcomes included: 1) fibrosis progression 2) hepatic decompensation 3) HCC and 4) LT-free survival. Predictors included longitudinal clinical and laboratory data. Machine learning methods were used to predict outcomes in 1 and 3 years.

**Results:**

The external cohort had a median age of 49.4 years (IQR 44.3–54.3); 61% were male, 80% white, and 79% had genotype 1. At presentation, 73% were treatment naïve and 31% had cirrhosis. Fibrosis progression occurred in 34% over a median of 4.9 years (IQR 3.2–7.6). Clinical outcomes occurred in 22% over a median of 4.4 years (IQR 3.2–7.6). Model performance for fibrosis progression was limited due to small sample size. The area under the receiver operating characteristic curve (AUROC) for 1 and 3-year risk of clinical outcomes was 0.78 (95% CI 0.73–0.83) and 0.76 (95% CI 0.69–0.81).

**Conclusion:**

Accurate assessments for risk of clinical outcomes can be obtained using routinely collected data across a heterogeneous cohort of patients with CHC. These methods can be applied to predict risk of progression in other chronic liver diseases.

## Introduction

Chronic liver disease remains one of the top 10 causes of death worldwide[1]. Patients with cirrhosis often require complex medical care including hospital admissions leading to an overall annual cost of over $2.5 billion in the United States alone.[2] The clinical course of chronic liver disease can be highly variable making risk stratification difficult. This has limited clinician’s ability to provide prognostic information and tailor clinical care to individual patients. Numerous prediction models have attempted to accurately assess risk of disease progression among patients with chronic liver diseases. Due to the reliance on classical forms of statistical analysis that restrict the number of predictor variables, these prior models primarily limited to baseline data have had only moderate accuracy in risk prediction.[3] We previously demonstrated that models that incorporate longitudinal data yield more accurate risk assessments by capturing serial results that reflect the dynamic nature of chronic liver disease progression.[4, 5]

Despite the marked improvement in efficacy and side effect profile of direct-acting antiviral agents (DAAs), chronic hepatitis C (CHC) remains a major public health problem due to the enormity of the worldwide disease burden, and the high proportion of chronically infected patients who have not been diagnosed or who do not have access to care.[6, 7, 8] Although multiple DAA combination regimens have been approved and competition has driven down the cost of treatment, many patients with early stage disease still encounter difficulty getting access to DAA therapy due to payers’ restriction of these medications only for patients with advanced fibrosis or cirrhosis.[9] Accurate prediction models of risk of disease progression in CHC patients who currently have early stage liver disease would be of benefit to both patients and clinicians to determine the risk of withholding treatment, even if temporarily, and to guide intensity of clinical monitoring. Ability to assess the risk of disease progression among patients who have been successfully treated would be of particular relevance to current clinical practice to help guide the level of monitoring of patients thereafter.

We previously demonstrated that using longitudinal data from the Hepatitis C Antiviral Therapy to Prevent Cirrhosis (HALT-C) trial database accurate risk assessments for both fibrosis progression and clinical outcomes could be obtained for patients with CHC using only data routinely obtained in clinical practice.[4, 5] However, all patients enrolled in the HALT-C trial had advanced liver disease (bridging fibrosis or cirrhosis) and had failed prior treatment; therefore, our prior prediction models may not apply to patients who have a broader range of liver disease severity and treatment exposure. In addition, only models for fibrosis progression and composite clinical outcomes were constructed in the previous study. The primary aim of this study was to validate longitudinal machine learning based prediction models for risk of fibrosis progression and clinical outcomes in a more heterogeneous cohort of patients including those with early stage disease and no prior treatment for CHC. Our secondary aim was to validate independent models for risk of hepatocellular carcinoma (HCC) alone and transplant-free survival among a heterogeneous patient cohort.

## Patients and methods

### Study population

The prediction models were initially constructed using 1050 patients in the HALT-C study. Patients enrolled in this trial had CHC with Ishak fibrosis score ≥3 on liver biopsy, a prior non-response to interferon (IFN) therapies, and no prior history of hepatic decompensation or HCC.[10] Patients in the HALT-C trial were randomized to maintenance therapy with pegylated-IFN or to no treatment for the next 3.5 years and were then followed without treatment (median follow-up over study duration 6.1 years).[10] External validation of our prediction models was performed using a retrospective cohort of adult patients (age ≥18) with CHC seen in the University of Michigan Health System (UMHS) hepatology clinic between January 1998 and June 2014. CHC in our UMHS cohort was defined as patients having a detectable HCV RNA in our electronic medical record. A proportion of patients with care established in the early time points of the study did not have an HCV RNA level documented in our electronic medical record (largely due to prior testing in outside facilities or decision not to initiate HCV treatment). In these cases, patients had a documented positive hepatitis C antibody (anti-HCV) and evidence of chronic liver disease without another etiology as assessed by their outpatient hepatologist (N = 224). Patients with <3 years of follow-up were excluded as our goal was to assess risk for intermediate term outcomes. Patients with hepatic decompensation, HCC, prior liver transplant (LT), and patients with hepatitis B virus or HIV co-infection at the time of initial evaluation were also excluded.

### Data collection

In the HALT-C cohort, liver biopsies were performed at baseline and repeated at 1.5 and 3.5 years. Patients were seen every 3 months during the randomized phase of the trial and every 6 months thereafter. During each visit blood tests were performed and patients were assessed for clinical outcomes. For our external validation cohort, demographic and baseline clinical characteristics including HCV genotype, HCV RNA level, HCV treatment history and response, as well as BMI and co-existing diabetes (defined based on clinical diagnosis in medical chart or prescription of an anti-diabetic medication) were abstracted using an electronic medical record search engine (EMERSE) that is designed to work with free text clinical documents in health records.[11] Results of baseline labs, imaging, histology and upper endoscopy were abstracted using electronic chart review. Baseline was defined as 6 months prior to or following the first hepatology clinic visit during the study period for imaging and lab results, and 12 months prior to or after initial clinic visit for histology and upper endoscopy. Longitudinal data of interest included serial labs, HCV treatment status and response, and follow-up imaging results. Specific labs abstracted included platelet count, international normalized ratio (INR), creatinine, albumin, total bilirubin, aspartate aminotransferase (AST), alanine aminotransferase (ALT), alkaline phosphatase and alpha-fetoprotein (AFP). Using these values, AST to platelet ratio index (APRI) and model for end stage liver disease (MELD) scores were calculated. Longitudinal laboratory results were exported from the electronic medical record with the assistance of data management experts in the Department of Pathology. Data was collected from date of initial clinic visit until June 30, 2014 or the achievement of an outcome. This study was approved by the University of Michigan Insitutional Review Board and granted a waiver of informed consent for HIPPAA authorization for retrospective chart audit and data collection.

### Outcomes and predictors

#### Primary and secondary outcomes

Our primary outcomes of interest were risk of fibrosis progression and risk of developing a composite clinical outcome. For the fibrosis progression outcome, only patients with at least 2 liver biopsies during the study period and with a baseline Ishak fibrosis score <5 were eligible to be assessed. Fibrosis progression was defined as an increase of ≥2 Ishak stages. For the composite liver-related clinical outcome, we assessed for the development of any of the following with the first event among these being captured as the time of outcome: hepatic decompensation (ascites including spontaneous bacterial peritonitis, variceal bleeding, or hepatic encephalopathy), HCC, liver transplant, or liver-related death. HCC was defined based on histology or radiology criteria according to the American Association for the Study of Liver Diseases (AASLD) guidelines.[12] We also performed several additional analyses to evaluate the performance of the composite clinical outcomes model in our external cohort based on achievement of sustained virologic response (SVR) and to account for the baseline presence or absence of cirrhosis. As a secondary aim, in this study we were also interested in assessing ability to predict risk of HCC alone and transplant-free survival (including all-cause mortality or need for liver transplant) alone.

#### Prediction variables

Only data that are routinely collected in clinical practice were used to construct the prediction models. Baseline predictors for the models included: age at initial presentation, gender, HCV genotype, HCV RNA, BMI, and history of diabetes. Serial labs analyzed included: platelet count, AST, ALT, total bilirubin, albumin, alkaline phosphatase, creatinine, AFP, INR, APRI, and MELD. In order to capture the extensive longitudinal data, for each laboratory predictor, we created 5 variables: mean, max, mean of differential, max of differential, and mean of acceleration. These variables were defined as follows: mean was defined as the mean of the observed values; max was the maximum of the observed values; mean of the differential was the mean of the difference between sequential observed values divided by the sequential observation time; max of the differential was the maximum of the difference between sequential observed values divided by the sequential observation time; and mean of acceleration was defined as the mean of the difference between sequential differential observed values divided by the difference between sequential differential observation time (Δẋ/Δt).

### Machine learning methods, model construction and external validation

#### Statistical methods

We used machine learning methods, specifically random forest (RF) analysis, to build prediction models because these methods are able to incorporate many predictor variables without compromising the accuracy of the risk prediction.[13, 14, 15] The details of this method are outlined in our prior publications, and are briefly summarized here.[4, 16, 17] Random forest is a decision tree-based ensemble statistical method that can build classification and regression prediction models, to identify baseline and longitudinal predictors associated with the development of an outcome. The random forest approach divides the cohort into two groups—x1 and x2 samples. The x1 sample is created using random sampling from the initial cohort. The x2 sample is composed of the unsampled data from the initial cohort, and typically includes about one-tenth of the initial cohort (10-fold cross validation). This process is repeated 50 times to get a precise point estimate. For each pairing, a decision tree is constructed using a random set of potential candidate variables for each split, and then validated using the x2 sample. As each tree is built, only a random subset of the predictor variables is considered as possible splitters for each binary partitioning. The predictions from each tree are used as “votes”, and the outcome with the most votes is considered the dichotomous outcome prediction for that sample. Using this method, multiple decision trees are constructed to create the final classification prediction model and determine overall variable importance. Accuracies and error rates are computed for each observation using the x2 samples, and then averaged over all observations. Because the x2 observations were not used in the fitting of the trees, they serve as cross-validated accuracy estimates. In random forest models, the criterion for splitting nodes for classification is the Gini index.[18] Variable importance identifies the most important variables based on their contribution to the predictive accuracy of the model. The most important variables are identified as those that most frequently result in early splitting of the decision trees. The final algorithms, consisting of 500 trees each, are not presented here for the sake of brevity.

Model performance was compared using the area under the receiver operating characteristic curve (AUROC) analysis and 95% confidence intervals (CI). The AUROC curves for the composite clinical outcomes models were used to identify optimal risk cut-offs to maximize the negative predictive value of the model in order to define a high-risk, “Clinical Progressors”, and a low-risk, “Clinical Non-Progressors”, group. Brier scores which capture both calibration and discrimination were also reported as an overall measure of model performance. The Brier score measures the mean squared difference between the predicted probability assigned to an outcome of interest and the actual outcome and takes on a value between zero and one. As such, the lower the Brier score, the better the prediction is calibrated with a Brier score of 0 representing a perfectly accurate prediction and a score of 1 being highly inaccurate. In order to assess the performance of our longitudinal model in the setting of missing data as may occur in the clinical setting, we used MissForest method of imputation for missing laboratory data.[19] MissForest is a non-parametric iterative method to impute missing data that unlike classical forms of imputation, is able to address missing data from multiple variable types (i.e. both continuous and categorical variables), and can cope with nonlinear relations and complex interactions. For each variable, MissForest fits a random forest on the observed part of the data and then predicts the missing pieces of data. The MissForest algorithm continues to repeat these steps until a stopping criterion is met or a maxium specified number of iterations is reached. As part of this imputation method, an estimate of the imputation error is provided. All machine learning methods were performed using the statistical language, R (version 3.0.2), with the statistical packages randomForest, missForest, Adaboost and gbm by Y.Z., B.L., and J.Z.[14, 15, 20] Descriptive and bivariate analyses were performed to analyze baseline characteristics and cumulative inicidence of outcomes of the cohorts using STATA 14 (StataCorp, College Station, TX). Chi-square tests and Fisher exact tests were used for categorical variables and t-tests were used for continuous variables. Variables with distributions that deviated from normality were compared using the Kruskal-Wallis test. Two-sided p values <0.05 were considered statistically significant.

#### Model construction and external validation

**Fig 1:**
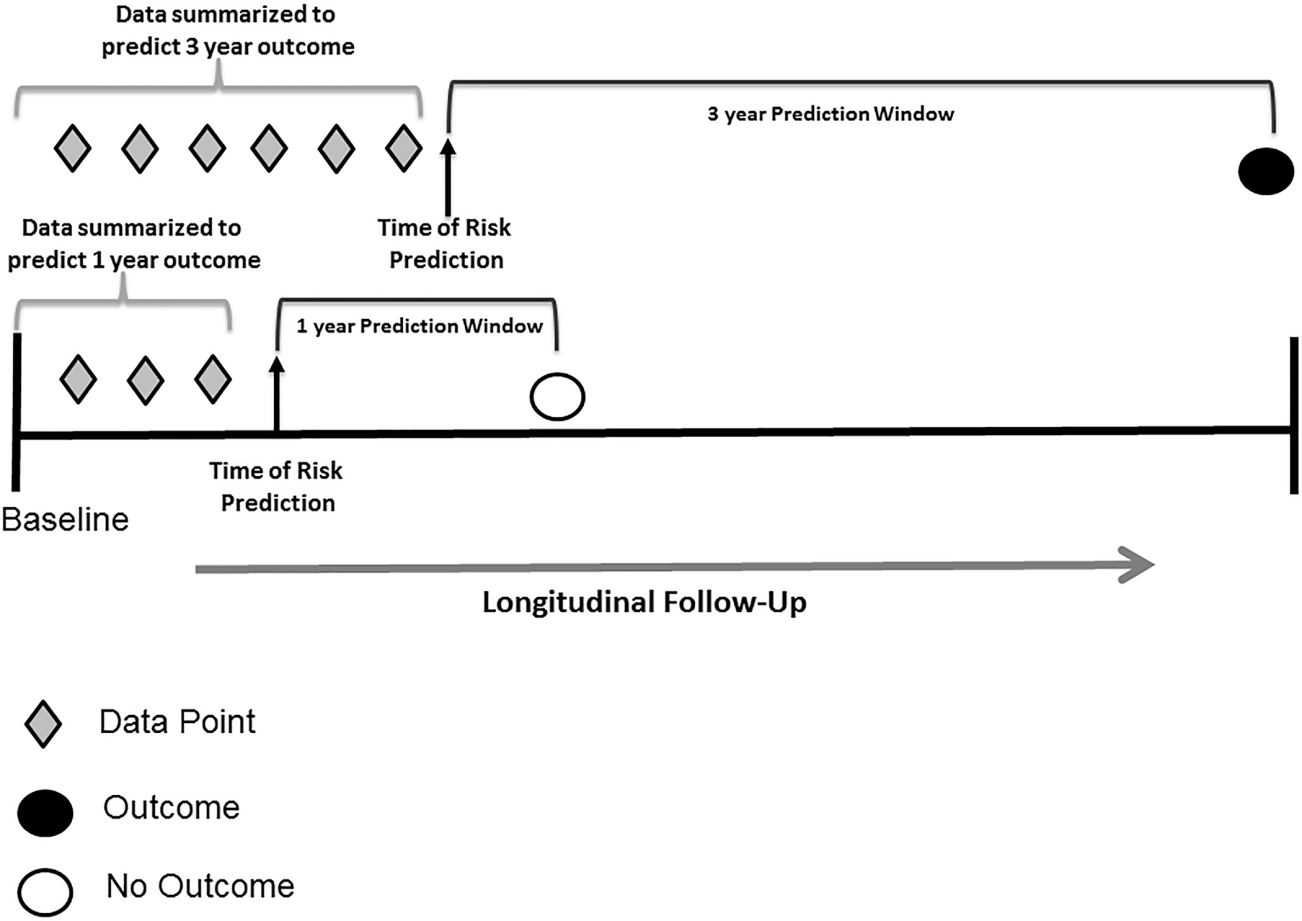
Risk prediction data assessment and timeframe.

For these analyses, we calculated the risk of an outcome as defined above using prediction time frames of 1 or 3 years in the future. For the risk prediction, we could then calculate the risk of an adverse outcome at a future time point of interest (prediction time) using all the data accumulated up to the time when a prediction is made (follow-up time) (**Fig 1**). We initially constructed new models for each outcome of interest using the HALT-C cohort among patients randomized to the non- interferon arm (in order to avoid potential medication effect on predictor labs). We constructed new models for fibrosis progression due to more concise predictor variables available in the UMHS cohort. A new model for composite clinical outcomes was built given the removal of change in CTP score compared to our prior model. We had not previously constructed machine learning longitudinal models for isolated clinical outcomes in our prior study, and thus these models were initially newly constructed witin the HALT-C cohort as well. We performed a 10-fold cross validation within these patients with each model contributing to the overall predictive accuracy (AuROC) of the model. We replicated this 50 times in order to obtain the mean and confidence interval for this AuROC. We then externally validated each of these models on all the data within the UMHS cohort.

## Results

### Baseline characteristics of the UMHS cohort

The baseline characteristics of patients in the UMHS cohort are displayed in **Table 1**. The median duration of follow-up was 6.9 years (IQR 4.5–10.5 years). Overall, the UMHS cohort was more diverse compared to patients enrolled in the HALT-C trial (**S1 Table**). The patients were primarily middle-aged (median 49.4 years, IQR 44.3–54.3) at the time of initial evaluation and 39% (N = 395) were female. Majority of the (80%, N = 636) patients was Caucasian and 79% (N = 755) had genotype 1 infection. In the UMHS cohort, 73% (N = 734) were treatment naïve. In total, 626 patients (62%) had a liver biopsy at baseline; of these, 18% (N = 97) had advanced fibrosis (Ishak 3–4) and 42% (N = 226) had cirrhosis (Ishak 5–6); and 14% had hepatic steatosis. Using a combination of biopsy and clinical information [histology or any two of the following: imaging showing cirrhosis (radiologist assessment as cirrhosis or any 2 of the following: nodular liver, ascites, splenomegaly, or abdominal collaterals), esophageal varices on EGD, or APRI >2], 315 patients (31%, N = 315) met criteria of having cirrhosis at initial presentation. Most patients were overweight but not obese (median BMI 28.2, IQR 25–32.4) and 14% (N = 147) had diabetes. During the study period, 485 patients (48.6%) were treated for HCV with 197 of these patients (41.5%) achieving SVR 12.

**Table 1:** Baseline characteristics of patients by outcome- UMHS cohort.

Variable	Overall Cohort (N = 1007)	Fibrosis Progression (N = 48)	No Fibrosis Progression (N = 92)	P value	Clinical Outcome (N = 226)	No Clinical Outcome (N = 781)	P value
***Demographics***							
**Age (median, IQR)**	49.4 (44.3–54.3)	47.5 (43.2–54.2)	48.7 (42.4–53.1)	0.77	51.1 (46.4–54.9)	49.1 (43.6–53.9)	**<0.001**
**Male**	612 (61%)	21(43.7%)	36 (39.1%)	0.59	147 (65%)	465 (60%)	0.13
**Race (N = 112;804)**				0.45			0.10
**White**	636 (80.1%)	33 (84.6%)	55 (75.3%)		143 (87%)	493 (79%)	
**Black**	127 (15.8%)	6 (15.4%)	16 (21.9%)		17 (10%)	110 (16%)	
**Asian**	18 (2.3%)	0 (0%)	2 (2.7%)		2 (1%)	16 (2%)	
***Clinical Characteristics***							
**HCV Genotype (N = 133;955)**				0.32			**<0.001**
**1**	755 (79%) No subtype: 292 1a: 255 1b: 208	37 (82.2%)	79 (89.8%)		158 (82%)	597 (78%)	
**2**	87 (9%)	3 (6.7%)	3 (3.4%)		5 (2.6%)	82 (10.7%)	
**3**	103 (10%)	6 (4.5%)	4 (4.5%)		28 (14.6%)	75 (9.8%)	
**Treatment Status (N = 1005)**				0.31			**<0.001**
**Naive**	734 (73%)	29 (60.4%)	62 (68.9%)		138 (61%)	596 (76%)	
**Experienced**	271 (27%)	19 (39.6%)	28 (31.1%)		88 (39%)	183 (24%)	
**Liver Biopsy**	626 (62%)	48 (100%)	92 (100%)		133 (21.2%)	493 (78.7%)	0.21
**Ishak Score**	N = 534			**0.004**	N = 115	N = 419	**<0.001**
**Ishak 0–2**	211 (39.5%)	34 (70.8%)	56 (60.8%)		17 (14.7%)	194 (46%)	
**Ishak 3–4**	97 (18%)	14 (29.2%)	36 (39.1%)		9 (8%)	88 (20.9%)	
**Ishak 5–6**	226 (42.3%)	N/A	N/A		89 (77%)	137 (32.7%)	
**Steatosis (N = 610)**	87 (14%)	8 (18.2%)	5 (6.9%)	0.06	23 (17%)	64 (13%)	0.25
**Cirrhosis**^**a**^	315 (31%)	N/A	N/A		147 (65%)	168 (21.5%)	**<0.001**
**BMI (median, IQR)**	28.2 (25–32.4)	27.4 ( 23.8–32.6)	28.0 (24.1–30.3)	0.86	30.1 (25.9–34.9)	28.1 (24.8–32)	**<0.001**
**Diabetes**	147 (14.6%)	5 (10.4%)	9 (9.8%)	0.91	55 (24.3%)	92 (11.7%)	**<0.001**
**APRI (median, IQR)**	0.96 (0.55–2.26)	0.71 (0.54–1.25)	0.7 (0.55–1.15)	0.98	2.92 (1.40–4.58)	0.78 (0.51–1.40)	**<0.001**
**HCV Treatment during Follow-up**	485 (48.1%)	28 (37.8%)	46 (62.1%)	0.35	66 (29.2%)	419 (53.6%)	**<0.001**
**SVR**	197(41%)	4 (14.3%)	12 (26.1%)	0.26	7 (10.6%)	190 (45.8%)	**<0.001**

^a^Cirrhosis based on biopsy or other clinical evidence

### Incidence of outcomes in the UMHS cohort

#### Fibrosis progression

In total, 140 patients in the UMHS cohort had at least 2 liver biopsies and an initial biopsy with Ishak fibrosis score <5 and were thus eligible to be assessed for the fibrosis progression outcome. At baseline biopsy, 90 patients (64%) had Ishak fibrosis stage 0–2 and 50 (36%) had Ishak fibrosis stage 3–4. The median duration between the biopsies was 3.9 years (IQR 2.1–5.7). Forty-eight (34%) patients had a ≥2-point increase in their Ishak fibrosis score over a median of 4.9 years (IQR 3.2–7.6). The baseline characteristics of patients who did and those that did not develop fibrosis progression or one of the composite liver-related clinical outcomes within the UMHS cohort are shown in **Table 1.** Comparative incidence of outcomes among the HALT-C cohort are provided in **S2 Table** for reference. Patients who had fibrosis progression did not appear to have specific distinguishing characteristics compared to those who did not have fibrosis progression.

#### Clinical outcomes

A total of 226 patients (22%) in the UMHS cohort developed one of the composite clinical outcomes. The median time to clinical outcome was 4.4 years (IQR 2.3–7.9) (**Table 2, S1 Fig**). Among the patients with a clinical outcome, the initial event was hepatic decompensation in 164 patients (72%) and HCC in 62 patients (28%). In total, 84 patients (8.3%) were diagnosed to have HCC after a median follow-up of 6.8 years (IQR 4.1–10.7), and 184 patients (18%) developed hepatic decompensation after a median follow-up of 4.3 years (IQR 2.0–6.8). Forty-one patients (4.1%) underwent LT after a median follow-up of 5.3 years (IQR 4.3–7.3). One hundred patients (9.9%) died of which 72 developed a preceding liver-related clinical outcome. 874 (86.8%) patients were alive without LT (**Table 2**). The 3-, 5-, and 7-year rates of overall survival were 99.8%, 98.3% and 96%, respectively; and the corresponding rates of transplant-free survival were 99.1%, 97.1%, and 93.9%, respectively. Patients who developed a clinical outcome were older, more likely to have genotype 3 infection, cirrhosis at baseline, prior HCV treatment, were less likely to undergo treatment over follow-up and to achieve SVR. Patients with a clinical outcome also had more pronounced metabolic disease with a higher baseline BMI and higher prevalence of diabetes.

**Table 2:** Cumulative incidence of outcomes in UMHS cohort.

Outcomes	Incidence Data
***Fibrosis Progression***	
**Serial Liver Biopsy**	140 (13.9%)
**Time interval between biopsy (median, IQR)**	3.9 yr (2.1–5.7)
**Fibrosis Progression**	48 (34%)
**Time to Fibrosis Progression (median, IQR)**	4.9 yr (3.2–7.6)
***Clinical Outcomes***	
**Composite Clinical Outcome**	226 (22%)
**Time to Composite Clinical Outcome (median, IQR)**	4.4 yr (2.3–7.9)
**First Liver-Related Clinical Outcome (N = 226)**	
**HCC**	62 (28%)
**Hepatic Decompensation**	164 (72%)
**Ascites**	95 (58%)
**Variceal Bleed**	15 (9%)
**Hepatic Encephalopathy**	26 (16%)
**Combination**	28 (17%)
**Hepatic Decompensation**	184 (18%)
**Time to Hepatic Decompensation** **(median, IQR)**	4.28 yr (2.03–6.8)
**HCC**	84 (8.3%)
**Time to HCC (median, IQR)**	6.68 yr (4.1–10.7)
**Liver Transplant**	41 (4.1%)
**Time to Liver Transplant (median, IQR)**	5.3 yr (4.3–7.3)
**Overall Mortality**	100 (9.9%)
**Time to Overall Mortality (median, IQR)**	8.0 yr (5.6–10.4)
**Transplant Free Survival**	874 (86.8%)
**Time to Liver Transplant or Overall Mortality (median, IQR)**	7.1yr (5.0–9.8)

### Predicting fibrosis progression

The model AUROCs for 1-year risk prediction was 0.62 (95% CI 0.50–0.75) among patients with complete data and 0.66 (95% CI 0.57–0.75) when imputed for patients with missing data. Given the overall small number of patients available to assess this outcome, we did not perform additional analyses to further characterize model performance for this outcome.

### Predicting composite liver-related clinical outcomes

**Fig 2:**
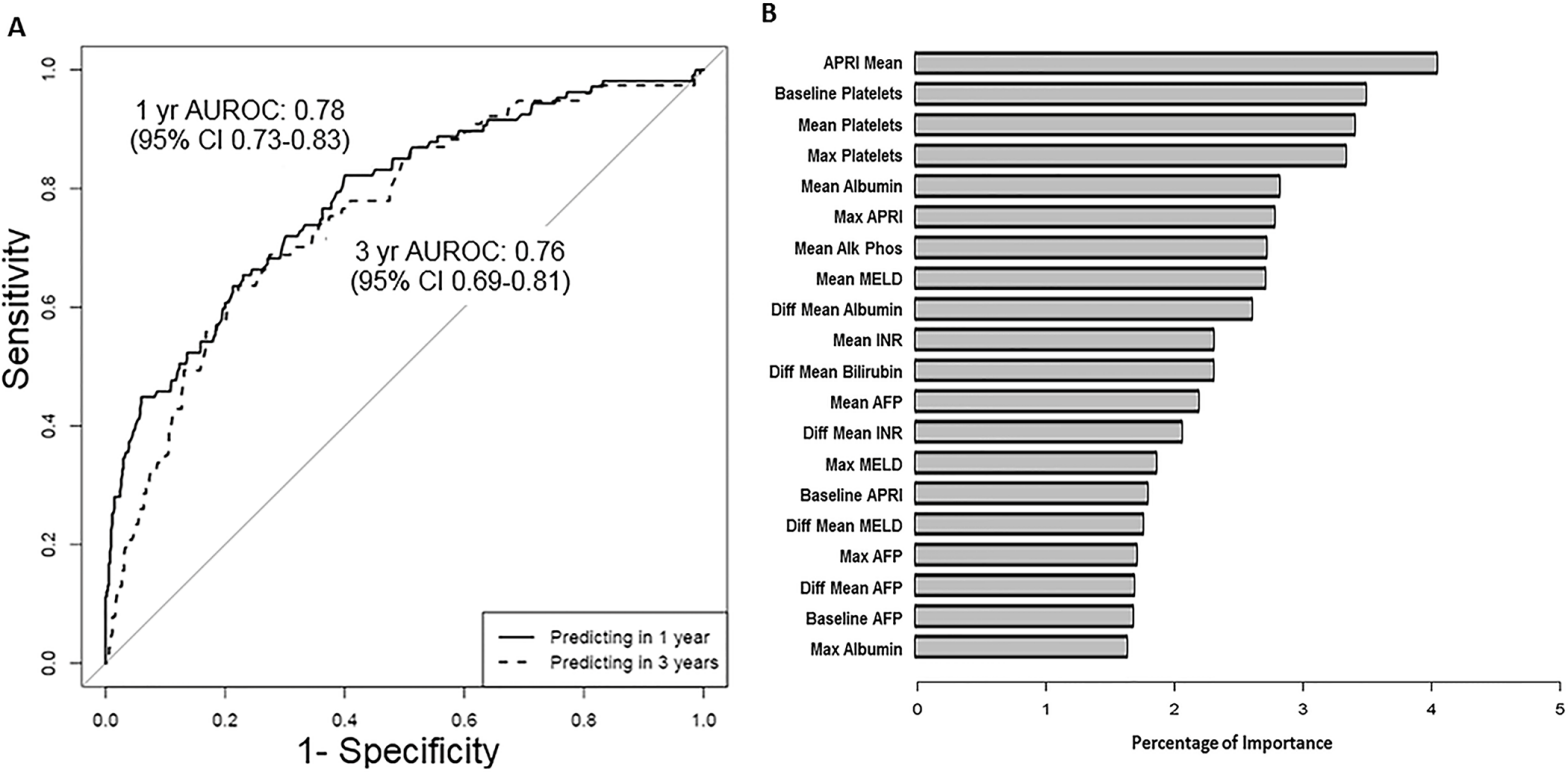
A) AUROC for 1 and 3-year Risk of Composite Liver-related Clinical Outcomes in UMHS Cohort; B) Variable Importance Graph for Predicting Composite Liver-Related Clinical Outcomes in UMHS Cohort.

Within the UMHS cohort, 31 patients (3%) developed a clinical outcome during the first year of follow-up and 80 patients (7.9%) developed a clinical outcome during the first 3 years of follow-up, and were excluded from prediction of 1- and 3-year clinical outcomes, respectively. We performed the primary analysis on the 642 and 595 patients who did not have any missing data for any of the predictor variables for 1 and 3-year risk predictions respectively. The AUROC results for the prediction model to differentiate high risk patients (“Clinical Progressors” who did develop a clinical outcome) from low risk patients (“Clinical Non-Progressors” who did not develop one of the composite liver-related clinical outcomes) in the UMHS cohort is displayed in **Fig 2A**. The AUROCs for models predicting composite liver-related clinical outcome at 1 year was 0.78 (95%CI 0.73–0.83) and at 3 years was 0.76 (95%CI 0.69–0.81). The variable importance graph for the model predicting composite clinical outcomes in the UMHS cohort is shown in **Fig 2B**. The most important independent variables for predicting composite liver-related clinical outcomes were as follows: mean APRI, baseline mean and maximum platelet count, and mean albumin. The analysis performed including patients with missing data for the predictors in the UMHS cohort yielded similar results (**S2 Fig**). The proportion of patients correctly classified as high vs. low risk and the associated Brier score in our external cohort is displayed in **Table 3.** For clinical outcomes, the 1 and 3-year risk prediction models had a sensitivity of 80% and 69%, a specificity of 62% and 65% and a negative predictive value (NPV) of 93% and 92% respectively.

**Table 3:** Misclassification table for composite liver-related clinical outcomes- UMHS cohort.

Clinical Progression								
Prediction Interval	Cut off	Clinical Progressors (1 year N = 191) (3 year N = 142)		Clinical Non-Progressors (1 year N = 773) (3 year N = 754)				
		Predicted Clinical Progression	Predicted No Clinical Progression	Predicted Clinical Progression	Predicted No Clinical Progression	Brier score	NPV	PPV
**1 Year**	0.458	153 (80.2%)	38 (19.8%)	294 (38.1%)	479 (61.9%)	0.344	92.6%	34.2%
**3 Years**	0.462	98 (69.1%)	44 (30.9%)	264 (35.1%)	490 (64.9%)	0.344	91.8%	27.1%

NPV, negative predictive value; PPV, positive predictive value.

In order to assess the effect of SVR on developing clinical outcomes, we performed additional analyses. 197 patients (19.5%) achieved SVR during follow-up. These patients were younger (median age 48.6 vs 49.6), and more likely to be Caucasian (87.9% vs 78.9%) and males (68.5% vs 58.9%). A higher proportion of patients who achieved SVR had genotype 2 infection (17.1% vs 7.1%) and were treatment naïve (85.8% vs 69.9%) without cirrhosis (74.7% vs 67.3%). Among the patients who achieved SVR during follow-up, only 7 (3.5%) developed one of the composite clinical outcomes compared to 27% in those who were not treated or did not achieve SVR. Among these 7 patients with outcomes, five had baseline cirrhosis. Five patients developed HCC and two had hepatic decompensation. In one of the 7 cases, the clinical outcome occurred prior to achieving SVR. We performed a separate subgroup analysis to assess the model performance for the composite clinical outcome among those who did versus those who did not achieve SVR. The AUROC for the composite clinical outcome model among those who achieved SVR was 0.96 (0.89–1.0) and 0.94 (0.84–1.0) for 1 and 3-year risk prediction respectively. Among patients who did not achieve SVR, it was 0.77 (0.72–0.82) and 0.74 (0.68–0.80) for 1 and 3-year risk prediction respectively (**S3 Table**). We also subdivided the overall cohort based on the stage of liver disease at the time of presentation to assess the effect of cirrhosis on model performance (cirrhosis vs. no cirrhosis using either biopsy or clinical criteria as defined above). The AUROC for 1 and 3-year risk of composite liver-related clinical outcomes among patients with cirrhosis at baseline (N = 315) was 0.70 (95%CI 0.62–0.78) and 0.69 (95% CI 0.60–0.78), and was 0.75 (95% CI 0.66–0.84) and 0.71 (95% CI 0.61–0.80) among patients without cirrhosis at baseline (N = 692).

### Predicting isolated clinical outcomes

**Fig 3:**
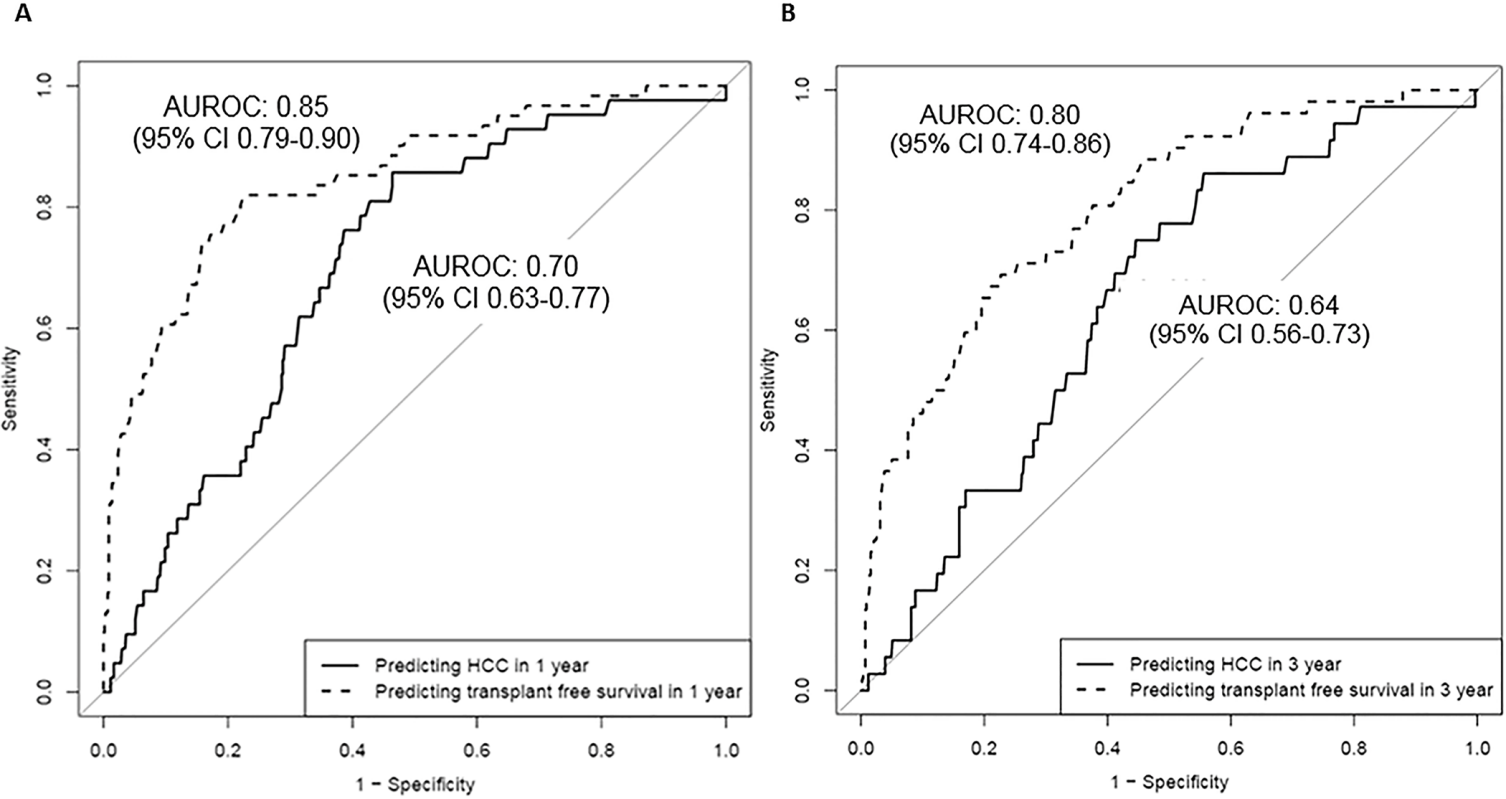
A). AUROC for 1-year Risk Prediction for Isolated Clinical Outcomes in UMHS Cohort. B). AUROC for 3-year Risk Prediction for Isolated Clinical Outcomes in UMHS Cohort.

The model performance for predicting risk of HCC alone at 1 and 3 years was less robust with AUROCs of 0.70 (95% CI 0.63–0.77) and 0.65 (95% CI 0.56–0.73), respectively in the UMHS cohort **(Fig 3A and 3B)**. When assessing transplant-free survival, the model performance was excellent with a 1 and 3-year AUROC of 0.85 (95% CI 0.79–0.90) and 0.80 (95% CI 0.74–0.86), respectively in the UMHS cohort **(Fig 3A and 3B)**. These models also retained their performance accuracy when using the entire validation cohort including patients with missing data for the predictors. A summary of model performances in the original HALT-C cohort and our external UMHS cohort is provided in **S4 Table** for reference.

## Discussion

Despite revolutionaly treatment advances, CHC remains a significant public health problem due to the global disease burden and the deficiencies in diagnosis and access to care and curative treatment. Given the nonlinear progression of CHC, clinical risk prediction for both short and intermediate term adverse outcomes is important if treatment delays are encountered. Obtaining accurate assessments of risk for disease progression and complications among patients who achieve SVR is also highly relevant to current clinical practice. Herein we demonstrate that highly accurate predictions for risk of adverse clinical outcomes can be made using only data obtained in routine clinical practice and across patients with diverse fibrosis stages, prior treatment exposures and response, HCV genotypes, demographics, and metabolic risk factors.

We previously constructed longitudinal models with high degree of accuracy in assessing risk of fibrosis progression and adverse clinical outcomes using the HALT-C database. The performance of our fibrosis progression models was less robust in the external cohort largely due to the small numbers of patients eligible for analysis as a result of the infrequency of serial liver biopsies in clinical practice. This suboptimal performance highlights the difficulties in assessing risk for earlier outcomes such as incremental increases in fibrosis stage among patients with early stage disease. In order to assess a larger number of patients at risk for fibrosis progression but who were lacking serial biopsies, future studies would benefit from incorporating non-invasive asessment of fibrosis that are now commonly used in clinical practice, e.g. liver elastography.

Our ability to accurately risk stratify patients for clinical outcomes was retained among this much more heterogeneous cohort however. Our short (1 year) and intermediate (3 year) term prediction models had robust AUROCs (0.78 and 0.76 respectively) with NPVs of 92–93%. This remained true independent of baseline cirrhosis. Interestingly, the model performance was better among patients without cirrhosis versus with cirrhosis, though this is likely is a reflection of the smaller number of patients with cirrhosis at baseline. We also demonstrated that our models remained accurate irrespective of SVR status, and in fact were more accurate among patients who achieved SVR. If validated among larger populations of patients who achieved SVR, application of these types of risk stratification models in clinical practice can help tailor monitoring post-SVR in this growing subgroup of patients. Notably, patients who developed a composite clinical outcome in our external cohort had a higher proportion of HCV genotype 3 infection and a higher prevalence of metabolic disease (higher BMI and more frequent diabetes).[21, 22, 23, 24] Optimizing the modifiable metabolic parameters may help mitigate risk of disease progression, a highly relevant opportunity given the increasing burden of co-existing hepatic steatosis among patients with HCV infection. Lastly, we also validated our new models for LT-free survival (AUROC 0.85 for 1 year and 0.80 for 3 years).

Accurate risk assessments for clinical outcomes across a heterogeneous patient population represent the major strength of our study. The model construction based on only data routinely collected in clinical practice also facilitates application of our models into clinical practice. Several limitations are to be noted including the lack of inclusion of patients with HIV or HBV co-infection and patients with less than 3 years of follow-up. Additionally, while this external cohort was more diverse than the HALT-C cohort, it continued to have a Caucasian male predominance and mainly comprised of genotype 1 infection. Our ability to predict risk of developing HCC alone remains suboptimal and may be due to the fact that variables reflecting severity of liver disease are insufficient to predict risk of HCC. This limitation reflects the ongoing need for further investigation into the pathophysiologic mechanisms driving onconeogenesis in patients with CHC, particularly as this risk persists among patients with cirrhosis despite the achievement of SVR.

In conclusion, we have successfully externally validated models for short and intermediate term risk of adverse clinical outcomes for patients with CHC among a diverse cohort of patients regardless of cirrhosis status and in those who did or did not achieve SVR. We found that patients with genotype 3 infection and those with metabolic co-morbidities had higher risk of clinical outcomes emphasizing the importance of development of effective DAA regimens for genotype 3 and prevention and reversal of metabolic abnormalities. Our models could also be applied to predict risk of clinical outcomes among patients with baseline cirrhosis who subsequently achieved SVR although the accuracy of our models in predicting HCC was low. From a health services standpoint, this statistical and clinical research approach can be applied more broadly to other forms of chronic disease in which risk stratification for adverse outcomes would be similarly germane.

## Supporting information

S1 Table Baseline characteristics of patients in HALT-C versus UMHS cohort. (DOCX)

S2 Table Cumulative incidence of outcomes in HALT-C xohort. (DOCX)

S3 Table Sub analysis of composite clinical outcome model AUROC according to achievement of SVR. (DOCX)

S4 Table Summary of model performance in HALT-C and UMHS cohorts. (DOCX)

S1 Fig Kaplan-meier survival curve demonstrating time to composite clinical outcome in UMHS cohort. (TIF)

S2 Fig AUROC 1 and 3-year risk prediction for composite liver-related clinical outcomes including patients with imputed data in UMHS cohort. (TIF)
