# Supplementary material for: Assessing risk of fibrosis progression and liver-related clinical outcomes among patients with both early stage and advanced chronic hepatitis C
Source: PLoS One. 2017 Nov 6;12(11):e0187344. doi: 10.1371/journal.pone.0187344 (PMC5673203; doi:10.1371/journal.pone.0187344)
Supplement: S1 Table — (DOCX) [file pone.0187344.s001.docx]

**Supplement Table 1. Baseline Characteristics of Patients in HALT-C versus UMHS Cohort**

| **Variable** | **HALT-C Cohort**  **(N=1050)** | **UMHS Cohort**  **(N=1007)** |
| --- | --- | --- |
| **Age (median, IQR)** | 49 (46-54) | 49.4 (44.3-54.3) |
| **Male** | 745 (71%) | 612 (61%) |
| **Race (% White)** | 752 (71.6%) | 636 (80.1%) |
| **% HCV genotype 1** | 980 (93.3%) | 755 (79%) |
| **Baseline Ishak Fibrosis Score Overall**  **(median, IQR)** | 4 (3-5) | 4 (2-6) |
| **Baseline Ishak Fibrosis Score 5 or 6** | 428 (40.7%) | 226 (42.3%) |
| **BMI (median, IQR)** | 29.1 (26.2-32.7) | 28.2 (25-32.4) |
| **Diabetes (%)** | 183 (17.4%) | 147 (14.6%) |
| **APRI (median, IQR)** | 1.09 (0.63-2.01) | 0.96 (0.55-2.26) |

APRI, AST to platelet ratio index; AST, aspartate aminotransferase; BMI, body mass index;

HALT-C, Hepatitis C Antiviral Therapy to Prevent Cirrhosis; HCV, hepatitis C virus; UMHS,

University of Michigan Health System
