# Supplementary material for: Assessing risk of fibrosis progression and liver-related clinical outcomes among patients with both early stage and advanced chronic hepatitis C
Source: PLoS One. 2017 Nov 6;12(11):e0187344. doi: 10.1371/journal.pone.0187344 (PMC5673203; doi:10.1371/journal.pone.0187344)
Supplement: S2 Table — (DOCX) [file pone.0187344.s002.docx]

**Supplement Table 2. Cumulative Incidence of Outcomes in HALT-C Cohort**

| **Outcomes** | **Incidence Data** |
| --- | --- |
| ***Fibrosis Progression*** | |
| **Serial Liver Biopsy** | 547 (52.1%) |
| **Time interval between biopsy (median, IQR)** | 3.85 yr (1.5-3.85) |
| **Fibrosis Progression (N=547)** | 152 (27.8%) |
| **Time to Fibrosis Progression (median, IQR)** | 3.85 yr (1.5-3.85) |
| ***Clinical Outcomes*** | |
| **Composite Clinical Outcome** | 242 (23.1%) |
| **Time to Composite Clinical Outcome (median, IQR)** | 3.7 yr (2.2-5.4) |
| **First Liver-Related Clinical Outcome** | |
| **HCC** | 74 (30.6%) |
| **Hepatic Decompensation** | 133 (54.9%) |
| **Liver Transplant** | 10 (4.1%) |
| **Liver-Related Death** | 25 (10.3%) |
| **Hepatic Decompensation** | 137 (13.0%) |
| **Time to Hepatic Decompensation (median, IQR)** | 3.27 (1.8-4.5) |
| **HCC** | 88 (8.4%) |
| **Time to HCC (median, IQR)** | 6.1 (4.1-6.9) |
| **Liver Transplant** | 86 (8.2%) |
| **Time to Liver Transplant (median, IQR)** | 4.1 (2.8-5.5) |
| **Overall Mortality** | 138 (13.1%) |
| **Time to Overall Mortality (median, IQR)** | 6.1 (4.6-6.9) |
| **Transplant Free Survival** | 839 (79.9%) |
| **Time to Liver Transplant or Overall Mortality (median, IQR)** | 4.27 (2.8-5.7) |
