# Supplementary material for: Assessing risk of fibrosis progression and liver-related clinical outcomes among patients with both early stage and advanced chronic hepatitis C
Source: PLoS One. 2017 Nov 6;12(11):e0187344. doi: 10.1371/journal.pone.0187344 (PMC5673203; doi:10.1371/journal.pone.0187344)
Supplement: S3 Table — (DOCX) [file pone.0187344.s003.docx]

**Supplement Table 3. Sub Analysis of Composite Clinical Outcome Model AUROC according to achievement of SVR**

|  | **UMHS** | | | |
| --- | --- | --- | --- | --- |
|  | **Complete Data**  AUROC  # with composite clinical outcome/  Total # available for assessment | | **Imputed for Missing**  AUROC  # with composite clinical outcome/  Total # available for assessment | |
|  | **1 year** | **3 year** | **1 year** | **3 year** |
| **With SVR** | 0.96 (0.89-1.0)  4/139 (2.8%) | 0.94 (0.84-1)  3/132 (2.2%) | 0.89 (0.79-0.99) 6/193 (3.1%) | 0.84 (0.70-0.98) 5/186 (2.6%) |
| **Without SVR** | 0.77 (0.72- 0.82)  103/503 (20.4%) | 0.74 (0.68-0.80)  74/463 (15.9%) | 0.77 (0.74-0.81)  185/771 (23.9%) | 0.73 (0.69-0.78)  137/710 (19.2%) |
