# Supplementary material for: Assessing risk of fibrosis progression and liver-related clinical outcomes among patients with both early stage and advanced chronic hepatitis C
Source: PLoS One. 2017 Nov 6;12(11):e0187344. doi: 10.1371/journal.pone.0187344 (PMC5673203; doi:10.1371/journal.pone.0187344)
Supplement: S4 Table — (DOCX) [file pone.0187344.s004.docx]

**Supplement Table 4. Summary of Model Performance in HALT-C and UMHS Cohorts**

|  | **HALT-C** | | **UMHS** | | | |
| --- | --- | --- | --- | --- | --- | --- |
|  |  |  | **Complete Data** | | **Imputed for Missing** | |
|  | **1 year** | **3 year** | **1 year** | **3 year** | **1 year** | **3 year** |
| Fibrosis Progression | 0.79  (0.77-0.81) | n/a | 0.62  (0.50-0.75) | n/a | 0.66  (0.57-0.75) | n/a |
| Composite Clinical Outcome | 0.83  (0.82-0.83) | 0.82  (0.81-0.83) | 0.78  (0.73-0.83) | 0.76  (0.69-0.81) | 0.78  (0.75-0.82) | 0.74  (0.69-0.79) |
| HCC | 0.67  (0.65-0.69) | 0.72  (0.71-0.74) | 0.70  (0.63-0.77) | 0.65  (0.56-0.73) | 0.67  (0.62-0.73) | 0.63  (0.56-0.69) |
| Transplant Free Survival | 0.82  (0.81-0.83) | 0.81  (0.80-0.82) | 0.85  (0.79-0.90) | 0.80  (0.74-0.86) | 0.79  (0.75-0.84) | 0.76  (0.72-0.81) |
|  |  |  |  |  |  |  |
