# Supplementary figures and images for: Assessing risk of fibrosis progression and liver-related clinical outcomes among patients with both early stage and advanced chronic hepatitis C
Source: PLoS One. 2017 Nov 6;12(11):e0187344. doi: 10.1371/journal.pone.0187344 (PMC5673203; doi:10.1371/journal.pone.0187344)

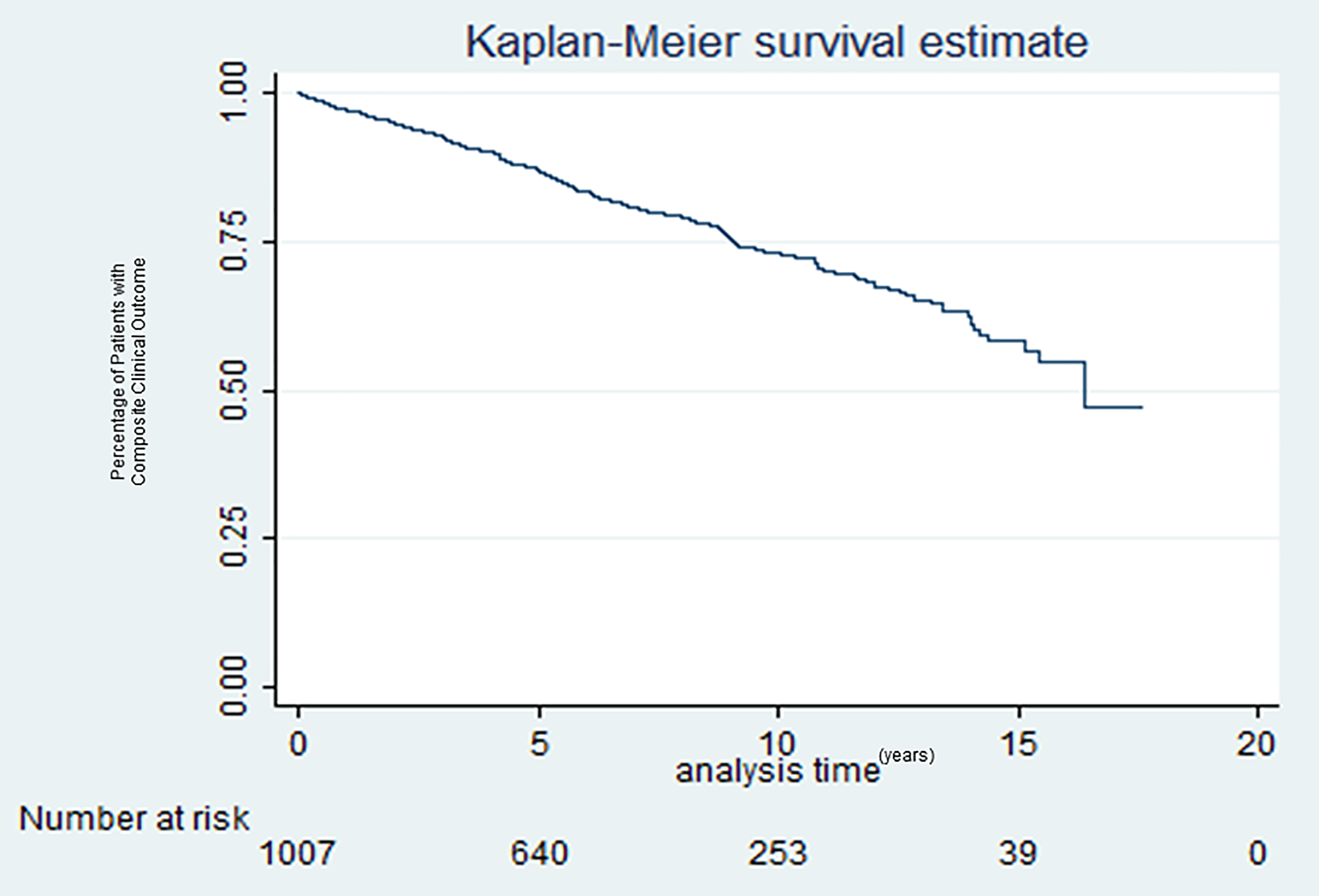

Supplement: S1 Fig — (TIF) [file pone.0187344.s005.tif]

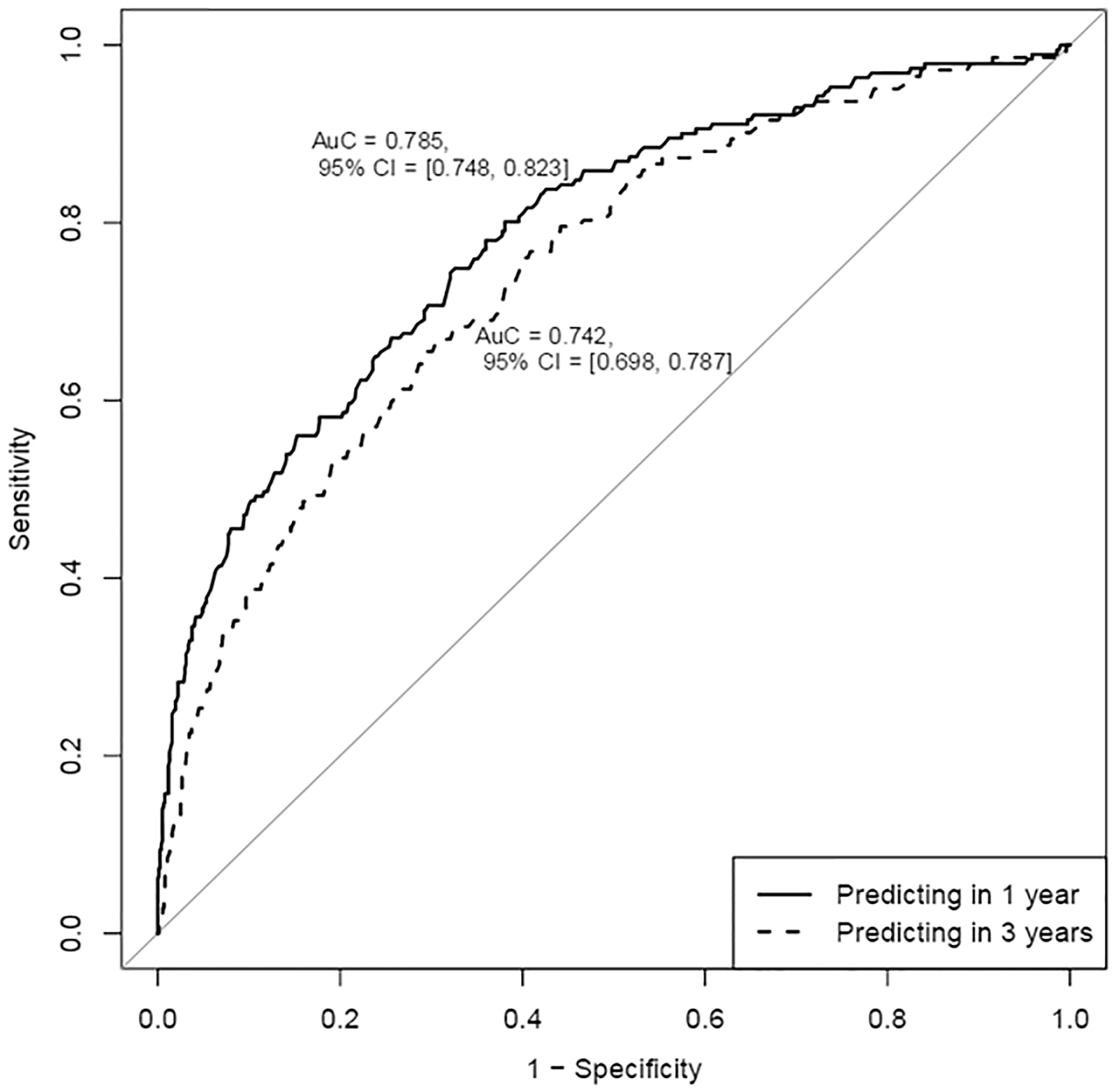

Supplement: S2 Fig — (TIF) [file pone.0187344.s006.tif]
